# Investigating Elder Abuse and Neglect in Diverse Refugee Communities in Greensboro, NC

**DOI:** 10.1093/geroni/igab046.1059

**Published:** 2021-12-17

**Authors:** S Sudha, Narayan Khadka

**Affiliations:** 1 University of North Carolina Greensboro, Greensboro, North Carolina, United States; 2 Institute for Peace and Harmony, Albany, California, United States

## Abstract

Elder abuse and neglect (EAN) comprises multiple dimensions, is experienced by about 10% of older adults in the U.S in diverse communities, and is severely detrimental to older adults’ (OA) health and wellbeing. However, documentation of EAN among refugee OA is greatly lacking as are services for these communities. Refugee OA are overall underserved members of marginalized communities. This paper reports on a community-engaged study to collect information and raise awareness of EAN among OA in 2 North Carolina refugee communities - Nepali-speaking Bhutanese and Congolese. Research partners included University researchers and community refugee-serving organizations. Surveys and focus group interviews were conducted. 17 Nepali-speaking Bhutanese and 13 Congolese filled out survey questions, including the Elder Abuse Suspicion Index. They participated in focus group discussions (FGDs), separately for men and women of each community. Survey results indicated EAN more among Congolese than Nepali-speaking Bhutanese. FGD results showed both communities prefer to depend on family members, and experience difficulties with language, transportation, and economic insecurity. No EAN was reported in the FGDs. In line with principles of community-engaged approaches, a capacity-building event to increase awareness of EAN was held, attended by 25 persons from the two communities. This study adds documentation on an under-researched area and marginalized communities. Action recommendations include disseminating culturally appropriate EAN information, strengthening English language and job skills and transportation options, encouraging cooperation across state, nonprofit, educational, and service organizations to address needs of older refugee adults.

